# *SmartFeeding4Kids*, an online self-guided parenting intervention to promote positive feeding practices and healthy diet in young children: study protocol for a randomized controlled trial

**DOI:** 10.1186/s13063-021-05897-z

**Published:** 2021-12-18

**Authors:** Ana Isabel Gomes, Ana Isabel Pereira, Tiago Guerreiro, Diogo Branco, Magda Sofia Roberto, Ana Pires, Joana Sousa, Tom Baranowski, Luísa Barros

**Affiliations:** 1 Faculty of Psychology, Research Center for Psychological Science (CICPSI), University of Lisbon, Alameda da Universidade, 1649-013 Lisbon, Portugal; 2 LASIGE, Faculty of Sciences, University of Lisbon, Campo Grande, 1749-016 Lisbon, Portugal; 3 Lisbon School of Medicine, Nutrition Laboratory, University of Lisbon, Avenida Professor Egas Moniz, 1649-028 Lisbon, Portugal; 4 Department of Pediatrics, USDA/ARS Children’s Nutrition Research Center, Baylor College of Medicine, 1100 Bates Street, Room 2038, Houston, TX 77030-2600 USA

**Keywords:** Parents, Preschool children, Dietary intake, Feeding practices, Online, Intervention, Randomized controlled trial

## Abstract

**Background:**

Caregivers’ influence on young children’s eating behaviors is widely recognized. Nutritional interventions that focus on the promotion of children’s healthy diet should actively involve parents, focusing on their feeding behaviors and practices.

**Methods:**

This work aims to describe the development and study protocol of the *SmartFeeding4Kids* (SF4K) program, an online self-guided 7-session intervention for parents of young (2–6 years old) children. The program is informed by social cognitive, self-regulation, and habit formation theoretical models and uses self-regulatory techniques as self-monitoring, goal setting, and feedback to promote behavior change. We propose to examine the intervention efficacy on children’s intake of fruit, vegetables, and added sugars, and parental feeding practices with a two-arm randomized controlled with four times repeated measures design (baseline, immediately, 3 and 6 months after intervention). Parental perceived barriers about food and feeding, food parenting self-efficacy, and motivation to change will be analyzed as secondary outcomes. The study of the predictors of parents’ dropout rates and the trajectories of parents’ and children’s outcomes are also objectives of this work.

**Discussion:**

The *SmartFeeding4Kids* program relies on technological resources to deliver parents’ self-regulation techniques that proved effective in promoting health behaviors. The study design can enhance the knowledge about the most effective methodologies to change parental feeding practices and children’s food intake. As a self-guided online program, *SmartFeeding4Kids* might overcome parents’ attrition more effectively, besides being easy to disseminate and cost-effective.

**Trial registration:**

The study was registered in ClinicalTrials.gov (NCT04591496) on October 19, 2020.

**Supplementary Information:**

The online version contains supplementary material available at 10.1186/s13063-021-05897-z.

## Introduction

Early childhood eating patterns are a growing public health concern, mainly because of low vegetable and fruit intake and high consumption of added sugars. Early establishment of healthy dietary patterns has long-term effects and is, therefore, a priority. It is recognized that parents should be the focus of actions to promote a healthy diet in young children [1], mainly through changes in their feeding behaviors. Parental feeding practices have been associated with children’s food preferences [2, 3], energy intake, and body mass index (BMI) [4–6]. Several programs for parents of young children support them in implementing alternative strategies to coercive or permissive practices [7]. However, systematic reviews showed that program efficacy studies rely mostly on children’s nutritional-related variables and rarely assess parental feeding practices as an outcome [8–10], specifically, children’s autonomy and self-regulation promotion practices [8].

Knowledge concerning the most effective ways to change parental feeding practices is still limited [11]. Self-regulation approaches have obtained positive results in changing parental behaviors [12] and engaging individuals in health behavior changes [13]. Web-based interventions may combine several methods to provide tailored information, prompt individual goal setting, promote reactive self-monitoring with individual feedback, and modeling [14]. eHealth interventions are also cost-effective, easy to disseminate, and have shown good acceptability and feasibility in involving parents to change children’s eating patterns [15–17].

This report aims to describe the *SmartFeeding4Kids* randomized controlled trial (RCT) protocol regarding the characteristics of the intervention, design, and procedures, and the outcome measures of the study. The intervention seeks to promote positive changes in parental feeding practices and their preschool children’s diet through self-regulation strategies and other behavior change techniques (BCTs). The RCT’s main objective is to examine the efficacy of *SmartFeeding4Kids* on children’s intake of fruit, vegetables, and added sugars, and parental feeding practices, comparing with a psychoeducational control condition throughout four assessment time points. We also aim to explore the role of parents’ (age, educational level, BMI, perception and concerns about the child’s weight, motivation to change, self-efficacy to promote the child’s healthy diet, perception of barriers regarding food and feeding) and children’s dimensions (age, sex, BMI, temperament) as predictors of parents’ dropout rates. Finally, we purpose to study the evolution of the children’s eating patterns, parental feeding practices, parental motivation to change, and feeding habits formation assessed by parents’ weekly monitoring throughout the intervention.

We hypothesize that after the *SmartFeeding4Kids* intervention, and in comparison with the control condition, parents will report [1] significant increase in structure and self-regulation promotion and reduction in ineffective control practices and (ii) significantly higher increase in children’s intake of vegetables and fruits and reduction of sugar-sweetened foods and beverages.

## Methods

### Study design

**Fig. 1:**
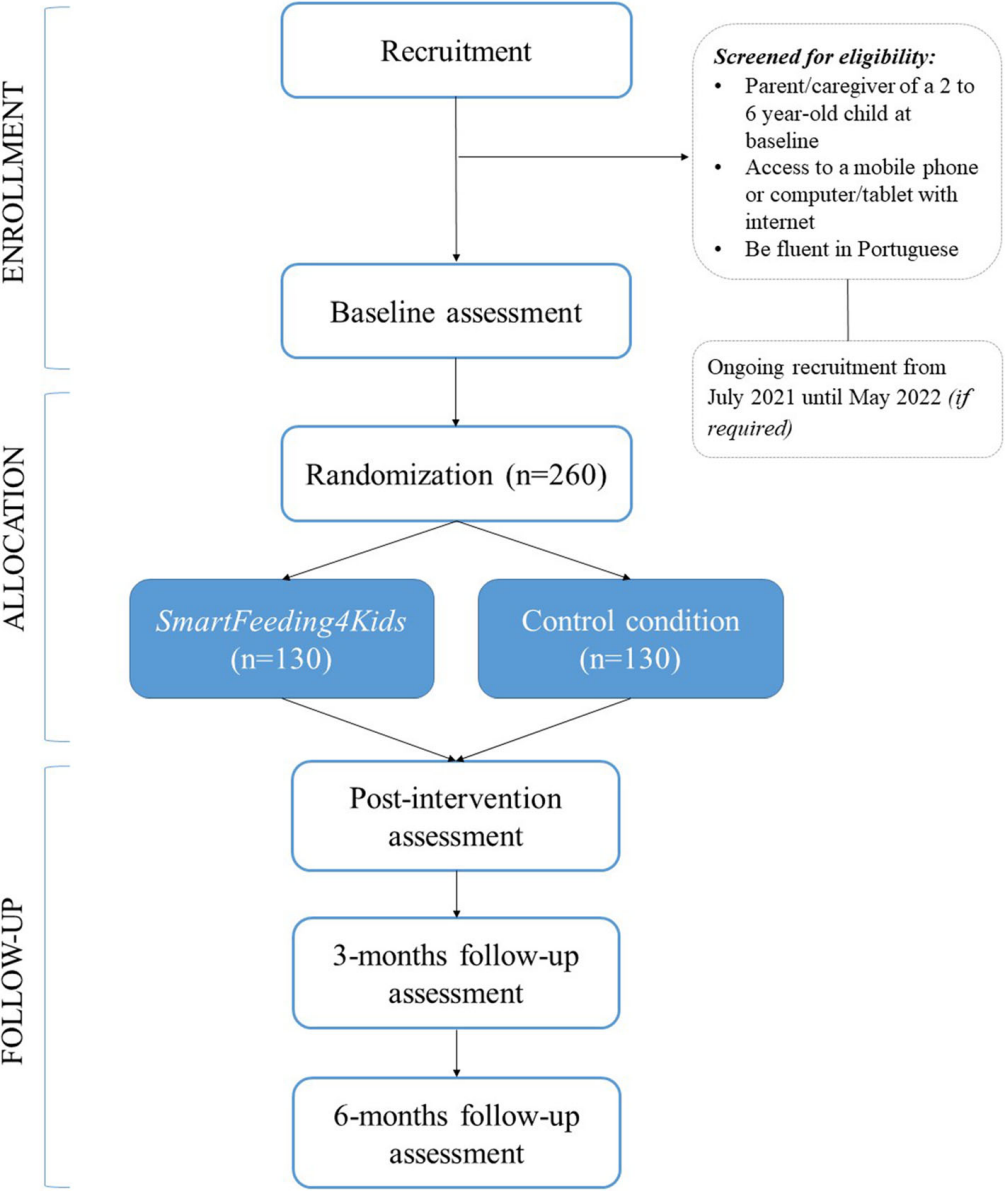
Flow diagram of the *SmartFeeding4Kids* RCT study

**Fig. 2:**
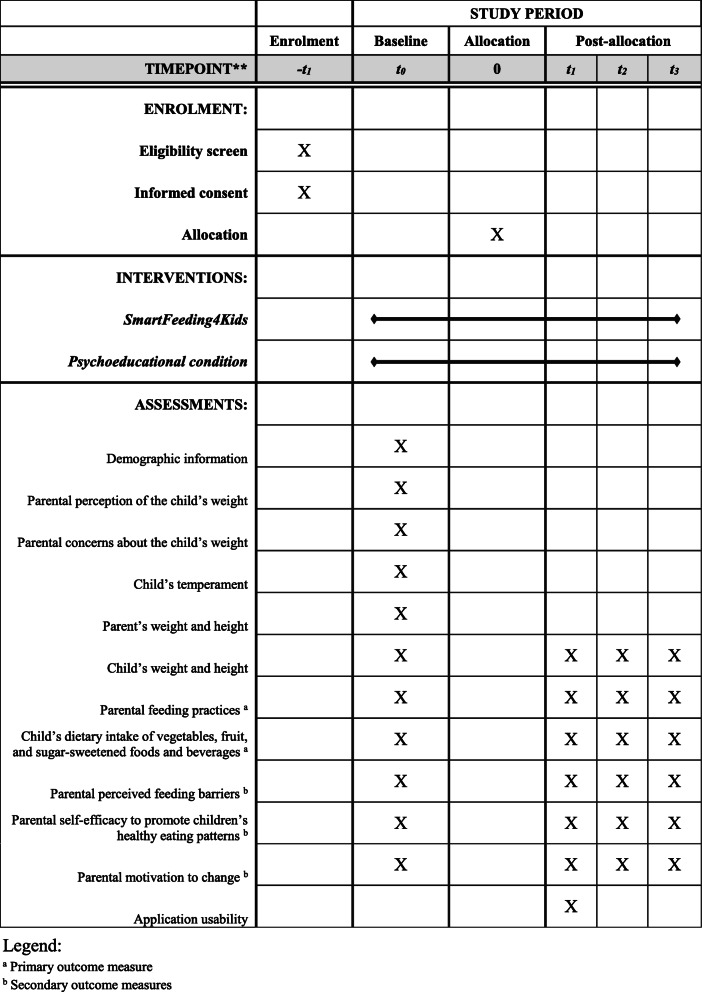
*SmartFeeding4Kids* RCT study schedule of enrolment, interventions, and assessments (according to SPIRIT guidelines)

To study the efficacy of the *SmartFeeding4Kids*, we adopted a randomized, controlled, superiority trial, with two-arm with four repeated measures design (Figs. 1 and 2). After baseline assessment, parents are randomly assigned to one of the two conditions: one experimental group (*SmartFeeding4Kids*) and one active comparator group. After finishing the program, parents complete the same evaluation protocol immediately and 3 and 6 months after the intervention. The study’s report follows the Consolidated Standards of Reporting Trials (CONSORT) statement [18] and the SPIRIT 2013 statement [19].

### Intervention

#### Theoretical framework

Three theoretical models support the *SmartFeeding4Kids* program: Social Cognitive Theory [20, 21], Model of Goal Directed Vegetable Parenting Practices [22], and Habit Formation Theory [23]. SCT recognizes that behavior acquisition and maintenance occur in a social context, in a reciprocal interaction among the behavior, personal expectations, learning from past experiences, observation of other’s behavior, and others’ reactions to individual’s actions (i.e., reciprocal determinism) [20]. When applying SCT to health promotion or disease prevention, Bandura [21] defined five core determinants of behavior adoption: knowledge of consequences of health behaviors; perceived self-efficacy to change and maintain health behaviors; outcome expectations about the costs and benefits of health actions; the goals that individuals set for themselves regarding health, the actions planned and the strategies implemented to achieve those goals; and the perceived facilitators and barriers in the achievement of those changes. Individual, behavioral, and environmental components of the SCT regarding parental feeding practices and promotion of children’s healthy dietary patterns will be addressed in the intervention through self-regulation strategies (e.g., goal setting, self-monitoring, and tailored feedback, modeling, social support, reinforcement) [20]. Previous research shows that self-regulation approaches have effectively changed parenting behaviors [12], guiding parents through a process involving setting individual goals and learning strategies to implement behavior changes leading to their child-related goals.

The Model of Goal Directed Vegetable Parenting Practices is an adaptation of the Model of Goal Directed Behavior developed to predict which individuals are more likely to engage in effective parenting practices to promote children’s vegetable intake [24]. This model highlights two important factors as central elements for intervention: parental perceived barriers and children’s feeding habits. Perceived barriers are the potential negative aspects of a health action that may discourage people from undertaking it [25, 26]. Parents often fail to implement effective parental feeding practices because they anticipate multiple obstacles. Therefore, identification and reduction of perceived barriers are proposed as critical components of food parenting interventions [22]. Habits are formed by repeating a behavior in a stable context, thus reinforcing a context-behavior association that triggers the habitual behavior, potentially without intention or effort [27]. As parental feeding involves behaviors occurring regularly and mostly in the same context, children’s feeding habits may be an efficient mechanism for maintaining effective parental feeding practices [22] and improving children’s dietary patterns [28]. In our intervention, we will guide parents in forming new feeding habits through three steps (i.e., habit setting and planning, establishing a routine, being persistent), helping them to achieve specific feeding habits, overcome possible obstacles, and reinforcing their achievements.

#### Experimental condition

The *SmartFeeding4Kids* program is a 7-session, self-guided intervention focused on promoting positive parental feeding practices to improve children’s dietary patterns. The components and contents of the *SmartFeeding4Kids* program are presented in Table 1. A detailed description of the BCTs included in each intervention condition [29] is available in Additional file 1.

**Table 1:** *SmartFeeding4Kids*: contents and components of the sessions

Session	Objectives	Contents and components	Between sessions
0. Invitation	Promote parents’ curiosity and interest in the program, and reflect on the reasons and advantages of participating in the study. Inform parents about the study, the program, and the tasks involved. Enhance parental motivation to commit to making positive changes in their feeding practices and the child’s dietary patterns.	Invitation. How important is your child’s diet to you? *SmartFeeding4Kids* program: How it works? Why is it worth participating? Informed consent form.	Records: Baseline assessment (Demographic information, Parental perception of the child’s weight, Parental concerns about the child’s weight, Child’s temperament, Parent’s weight and height, Child’s weight and height, Parental feeding practices, Parental perceived feeding barriers, Parental self-efficacy to promote children’s healthy eating patterns, Parental motivation to change). Includes monitoring of the child’s food intake (24-h food recall, 3 days).
1. How healthy is my child’s diet?	Increase parental knowledge about a healthy diet, formation of food preferences in childhood, and consequences associated with consuming healthy and unhealthy foods. Increase parental knowledge about specific dietary guidelines for preschool children’s vegetables, fruit, legumes, and sugar-sweetened foods and beverages intake (frequency and portion sizes). Help parents measure adequate food portions with the child’s hand. Foster parental self-regulation and self-efficacy to achieve changes in children’s food intake according to their needs.	First informative feedback regarding the child’s food intake: vegetables, fruit, legumes, and sugar-sweetened foods and beverages. What is a healthy diet? All children are different: how to respect children’s eating needs? Children and adults portion sizes. Guidelines for vegetables, fruits, and legumes intake: 3 + 2 + 1! Using the child’s hand to measure food portions. Sweet foods: innate preferences and habits. Types of sugar and foods with added sugar. Guidelines for sugar-sweetened foods intake: only on party days! How am I doing? Evaluative feedback about the child’s vegetable, fruit, legumes, and sugar-sweetened foods and beverages intake. Summary of the main messages of the session. Recipes for healthy foods. Goal setting (choose two goals for the child’s food intake). Degree of motivation to accomplish the chosen goals.	Records: Monitoring of the child’s food intake (24-h food recall, 1 day) Prompts: 3 (vegetables) + 2 (fruit) + 1 (legumes)! Remember your goals: (…)
2. All about feeding practices	Increase parental knowledge about the adverse effects of pressuring children to eat and offer foods as a reward. Increase parental knowledge about alternative positive feeding practices to increase children’s interest and acceptance of healthy foods Help parents to identify child-related barriers regarding food refusal and how to overcome them using positive feeding practices. Foster parental self-regulation and self-efficacy to achieve changes in children’s food intake and parental feeding practices according to their needs.	Am I achieving my goals? Weekly informative feedback (child’s food intake). Reinforcement and encouragement. Let’s review… Summary of the last session. What are parental feeding practices? First informative feedback regarding the feeding practices (pressure to eat, food as a reward, exposure to healthy foods, offering healthy food options, self-regulation teaching, modeling). What are pressure to eat and food as a reward? Examples of negative statements. Negative consequences of those practices for the child’s dietary intake and food preferences. So, what can I do instead? Introduction of the golden rule: *Parents decide what, when, and how the child eats; the child decides whether and how much they eat.* Alternative positive feeding practices to deal with child-related barriers (e.g., my child dislikes vegetables, my child is a picky eater). Instructions on how to apply these feeding practices, with examples of positive statements. How am I doing? Evaluative feedback regarding feeding practices (pressure to eat, food as a reward, exposure to healthy foods, offering healthy food options, self-regulation teaching, modeling). Quiz (problem-solving activity): three vignettes about a child’s refusal to eat and use of food as a reward, choosing the strategies/practices most suitable to deal with each situation, with feedback. Summary of the main messages of the session. Goal setting (choose two goals regarding feeding practices). Degree of motivation to accomplish the chosen goals.	Records: Monitoring the child’s food intake (24-h food recall, 1 day) and two parental feeding practices (max.) related to the goals chosen in the session Prompts: This is the correct order: parents serve, the child decides! Remember your goals: (…)
3. All about feeding practices [2]	Increase parental knowledge about the adverse effects of unhealthy food restriction and emotional feeding. Increase parental knowledge about alternative positive feeding practices to regulate children’s intake of sugar-sweetened foods or excessive amounts of food. Help parents identify child-related barriers regarding increased food ingestion or high preference for sugar-sweetened foods and beverages and how to overcome them using positive feeding practices. Foster parental self-regulation and self-efficacy to achieve changes in children’s food intake and parental feeding practices according to their needs.	Am I achieving my goals? Weekly informative feedback (child’s food intake and targeted feeding practices). Reinforcement and encouragement. Let’s review… Summary of the last session. First informative feedback regarding feeding practices (emotional feeding, food restriction, permissiveness, limitation of unhealthy food availability, self-regulation prompting). What are restriction and emotional feeding? Examples of negative statements. Negative consequences of those practices for the child’s dietary intake and food preferences. So, what can I do instead? Revision of the golden rule: *Parents decide what, when, and how the child eats; the child decides whether and how much they eat.* Alternative positive feeding practices to deal with child-related barriers (e.g., my child loves sodas, my child eats a lot). Instructions on how to apply these feeding practices, with examples of positive statements. Am I doing well? Evaluative feedback about feeding practices (emotional feeding, food restriction, permissiveness, limiting unhealthy food availability, self-regulation prompting). Quiz (problem-solving activity): three vignettes about a child’s food requests and emotional feeding, to choose the strategies/practices most suitable to deal with each situation, with feedback. Summary of the main messages of the session. Goal setting (choose two goals regarding new feeding practices). Degree of motivation to accomplish the chosen goals.	Records: Monitoring the child’s food intake (24-h food recall, 1 day) and four parental feeding practices (max.) related to the goals chosen in the session Prompts: Learn to enjoy, enjoy eating: no pressuring and no prohibiting! Remember your goals: (…)
4. Barriers: how to keep going?	Help parents identify parent-related and context-related barriers and how to overcome them using positive feeding practices. Foster parental self-regulation and self-efficacy to achieve changes in child’s food intake and parental feeding practices according to their needs.	Am I achieving my goals? Weekly informative feedback (child’s food intake and targeted feeding practices). Reinforcement and encouragement. Let’s review… Summary of the last session. First informative feedback regarding parent-related and context-related barriers. Parent and context-related barriers: What are the main challenges? Examples of obstacles related to parents’ food preferences, lack of cooking skills, others’ offering treats, and cost of healthy foods. So, what can I do instead? Alternative positive feeding practices to deal with parent and context-related barriers. Instructions on how to apply these feeding practices, with examples of positive statements. Quiz (problem-solving activity): three vignettes about parent and context-related barriers, to choose the most suitable strategies/practices to deal with each situation, with feedback. Summary of the main messages of the session. Identification of the most challenging child, parent and/or context-related barriers, and planning alternative positive strategies. List of previous goal setting. Motivation to accomplish the chosen goals.	Records: Monitoring the child’s food intake (24-h food recall, 1 day) and four parental feeding practices (max.) related to the goals chosen in the session Prompts: Be a good role model for your child! Remember your goals: (…)
5. Keep the good habits!	Increase parental knowledge about the formation of feeding habits. Help parents identify new feeding habits to introduce during mealtimes, make plans based on positive parental practices and overcome common obstacles. Help parents establish a routine and be persistent in keeping with it daily. Foster parental self-regulation and self-efficacy to achieve changes in children’s food intake, parental feeding practices, and feeding habits according to their needs.	Am I achieving my goals? Weekly informative feedback (child’s food intake and targeted feeding practices). Reinforcement and encouragement. Let’s review… Summary of the last session. Feeding habits: What is a habit? How are habits formed? How to transform a behavior into a habit? Five steps to form a new feeding habit: choose a behavior in response to a context cue, evaluate the behavior’s automaticity, make a plan, establish a routine and be persistent. Summary of the main messages of the session. Goal setting (choose two goals regarding new feeding habits). Degree of motivation to accomplish the chosen goals.	Records: Monitoring the child’s food intake (24-h food recall, 1 day), two parental feeding practices (max.), and two feeding habits related to the objectives chosen in the session Prompts: Best to bend while it is a twig! Healthy eating habits are formed during childhood. Remember your goals: (…)
6. Choose positive practices!	Reinforce parental self-regulation and self-efficacy to sustain changes in children’s food intake, parental feeding practices, and feeding habits.	Am I achieving my goals? Weekly informative feedback (child’s food intake and targeted feeding practices and habits). Reinforcement and encouragement.	Records: Monitoring the child’s food intake (24-h food recall, 1 day), two parental feeding practices (max.), and two feeding habits related to the goals chosen in the session Remember your goals: (…)
7. Stay strong!	Reinforce parental self-regulation and self-efficacy to sustain changes in children’s food intake, parental feeding practices, and feeding habits.	Am I achieving my goals? Weekly informative feedback (child’s food intake and targeted feeding practices and habits). Reinforcement and encouragement.	Records: Monitoring the child’s food intake (24-h food recall, 1 day), two parental feeding practices (max.), and two feeding habits related to the objectives chosen in the session Remember your goals: (…)

In the experimental condition, all sessions share a similar structure regarding the proposed tasks. The central theme is introduced, and descriptive feedback (i.e., provision of information about participant’s current behavior performance) regarding child’s food intake or parental feeding practices discussed in the session is shown. Parents then watch specific interactive content related to the session’s theme (e.g., information about nutritional guidelines for young children, effective and ineffective feeding practices, or how to form a new feeding habit). Parents can choose specific feeding challenges and barriers to learn more about how to overcome them. Evaluative feedback (i.e., evaluation of participant’s behavior performance according to specific guidelines and cut-off points) about the behaviors discussed in the session is presented and, in some instances, is followed by a quiz (problem-solving activity). Finally, parents are invited to choose two specific goals from a list of objectives tailored to their baseline assessment results [see Additional file 2 for a detailed description about outcome measurements and cut-off points used to tailor available goals in the sessions]. The degree of motivation to achieve the chosen goals (e.g., importance, confidence, readiness) is assessed through three subjective rating scales. From session 2 onwards, parents start to see their evolution regarding the child’s food intake and feeding practices and are informed whether they achieved their goals on specific behaviors during the week (i.e., comparative feedback) before the main theme is addressed. In the booster sessions (6 and 7), parents continue to monitor their practices and children’s food intake and receive feedback about their goals’ achievement. At the end of the last session, parents receive final feedback about their evolution throughout the program.

Between sessions, parents are asked to complete a 24-h food recall regarding children’s food intake on a day of their choice and answer questions about parental practices and feeding habits related to the goals chosen in each session. These records are available 3 days after completing each session and are asked to be accomplished during the following week. A new session is only accessible once parents have seen the last session’s contents and performed the tasks proposed between sessions. During this time, parents also receive a notification to remember the core message and the session’s goals [30]. Participants are rewarded with points and badges when specific targets are reached (e.g., when a selected goal is reached, when the feedback shows progress in performing a specific behavior compared with the last session), or when completing specific tasks (e.g., see a specific content).

#### Control condition

Parents assigned to the active comparator condition receive the same information as the experimental conditions regarding nutritional guidelines for young children and effective/ineffective parental feeding practices. However, this control group does not have access to interactive activities (e.g., quizzes) or self-regulation strategies during and between sessions (e.g., tailored feedback, goal setting, monitoring of the child’s food intake, and parental feeding practices).

#### Development of the *SmartFeeding4Kids* app

We designed and developed an application in Portuguese, the *SmartFeeding4Kids app*, to deliver all the intervention components and manage the trial. It can be used on web browsers or as an app on a mobile device and includes seven sessions that cater components from randomization, onboarding, 24 h food recall, delivery of information, personalized feedback to data collection (e.g., study questionnaires), and logging. Parents are guided through the program with new contents unblocked depending on the previous step and time.

**Fig. 3:**
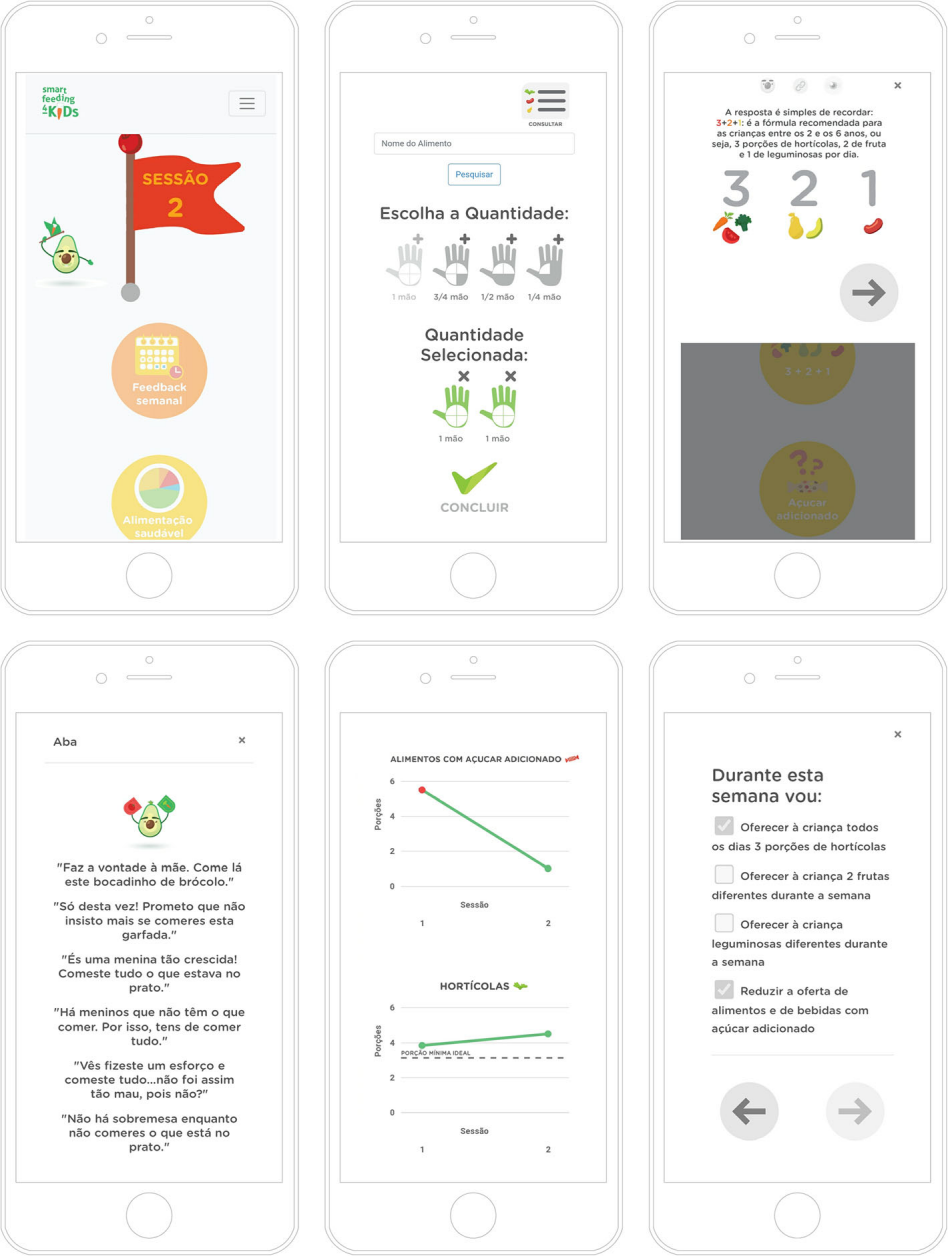
*SmartFeeding4Kids* *app* features (from the upper left edge to the lower right edge of the figure). (1) General view of the program: sessions’ sequence. (2) Child’s 24-h food intake: selection of the number of portions eaten by the child in that day, regarding a specific food/beverage. (3) Nutritional information content (session 1). (4) Aba’s intervention: examples of verbalizations regarding ineffective feeding practices (e.g., pressure to eat). (5) Evaluative feedback: information about the number of portions of sugar-sweetened foods/beverages and vegetables eaten by the child in the day(s) recorded. (6) Goal setting: list of theme-related goals proposed to parents at the end of session 1

The application was designed to be appealing and engaging. The visual design includes lively colors and several professional drawings of vegetables and fruits as characters (Fig. 3). Information boards are presented as slideshow animations where these characters give textual feedback illustrated with carefully designed visualizations. *Aba*, an avocado avatar, welcomes and accompanies parents in the application, guiding, informing, and motivating them through the sessions. Charts are presented to illustrate each session’s performance based on food diaries. To further fuel parents’ engagement, badges are given when they complete pre-defined achievements. Examples of badges are finishing a session, interacting with content, and daily logins, among others. In addition, to promote discoverability, we have introduced hidden rewards as special badges that are not visible before being won (e.g., a badge for filling the food recall meals along the day instead of all at the same time). Notifications and reminders are automatically sent via email to congratulate users for their performance, remind them of pending tasks, and announce new content.

The application was iteratively co-designed within a team of psychologists, engineers, designers, and a nutritionist. Starting from the program sessions and elements, the team went from discussing low-fidelity prototypes to iterating over high-fidelity increasingly functional ones in a process that lasted over a year. A workshop was conducted with psychologists and nutritionists to create a set of personas and daily scenarios that guide and question the design decisions of the platform, particularly regarding the engagement of the participants and dealing with the program’s demand. Along with several iterations among the team and a pilot study with 12 participants, the application was fine-tuned to improve usability and adherence. This process was pivotal to adapt the onboarding process, making *Aba* (the avatar) more pervasive in the application and adjusting the rewarding mechanisms to be balanced (e.g., number and type of badges and respective points awarded). Most of the iterations were done over how each session should be presented, with food recall being the most demanding one to make usable.

### Participant recruitment and eligibility criteria

Recruitment will be conducted out nationally; participation is open to all parents of 2 to 6-year-old children living in Portugal. Information about the program and how to participate in the study will be shared through social networks (e.g., Facebook, Twitter, Instagram) and online groups attended by parents. A brief multimedia presentation about the intervention will be disseminated in parents’ meetings at collaborating local childcare facilities. We will also contact health professionals in primary health care centers and pediatric consultations to ask them to disseminate the intervention among parents they think could benefit from the program’s participation.

Participants are eligible to participate if (a) they are a parent/caregiver of one 2- to 6-year-old child at baseline (if the parent has two children in this age group, the parent is instructed to focus on the child that raises more concerns about their health habits, as reference); (b) have a mobile phone or computer/tablet with access to the internet; and (c) are fluent in Portuguese.

### Power and sample size

Statistical power analysis was performed for sample size estimation based on published data regarding this topic using G*Power 3.1.9.2. For our repeated measures within-between group comparison study, we used Cohen’s *f* criteria and considered a small effect size of 0.15 [8, 31], an alpha equal to 0.05, and power of 0.80 for a two-group with four repeated measures design (with a moderate correlation pattern between time measurements of 0.35). The sample size needed is approximately 130 participants (i.e., 65 parents in each of the two-arm groups), but the total sample size was adjusted to account for a dropout rate of 50% [32, 33]. Therefore, the sample will be collected until 130 participants in each group (*N* = 260) enroll in the study. Our proposed sample size will also be adequate for multilevel modeling with level 1 repeated measures nested within level 2 individuals, by assuring a minimum of 50 individuals required for level 2 [34].

### Randomization process

The random sequence generation process and the participants’ allocation to the intervention conditions will be run automatically by the online platform. After completing the baseline assessment, parents will be randomized and allocated to one of the conditions through an automated computerized randomization program, with an equal allocation ratio (1:1); the online intervention will be immediately available for parents after the allocation.

Parents will be blinded to which condition they were assigned during the trial. Participants are aware that the two interventions will be run and compared, and the tasks involved in each will be summarily reported, but they are not informed about the study’s hypothesis. The use of an active comparator intervention as a control condition will reinforce the blinding mechanism: parents will receive a similar intervention regarding the sequence of contents, duration, and the information available, but without the behavior change techniques intended to change parents’ and children’s behaviors (i.e., knowledge-based intervention). Researchers will be blinded to group allocation. Because of the intervention’s nature, which is programmed to be delivered automatically by the online platform according to the parent’s pace and answers, researchers do not participate directly in the intervention and cannot change its course.

### Study procedures

Once parents access the site and complete the registration, they are invited to identify their reasons for participating in the intervention and receive information about the program’s characteristics and the feeding situations in which the intervention can support them. Then, parents are asked to read the online informed consent form, including detailed information about the program (e.g., study objectives, eligibility criteria, information about the study groups and tasks required in each one, random allocation procedures, data collected during the study). If parents agree to participate, the consent form is sent automatically to the participant’s email, and they are directed to the baseline assessment protocol, to be completed within 2 weeks. Parents are also asked to record their child’s food and portion intake for 3 days (two weekdays and one weekend day). The days to perform the 24-h food recalls are randomly chosen by the app, and parents receive a notification the day before. After completing these tasks, each parent is allocated automatically to one condition, and session 1 is released. At the end of the program, parents receive a notification to fill the post-intervention evaluation protocol within a maximum of 15 days. A similar procedure occurs 3 and 6 months after the intervention.

There will be no special criteria for discontinuing or modifying allocated interventions. Parents of children with any medical condition that may affect dietary behavior or growth (e.g., food allergies or intolerances, chronic health conditions) or receiving other professional counseling can participate in the program. They are encouraged to discuss any doubts about the recommendations provided by the program with the child’s assistant, to better suit their child’s condition; the healthcare professional can contact the investigators through the project email.

Several procedures aim to facilitate sample maintenance throughout the program. The platform sends automatic emails when parents take longer than expected to complete the tasks, reminding them about the timings and goals chosen in the sessions, giving new opportunities, or motivating parents to login into the platform. Parents can send an email if they experience any technical difficulty. The IT team will monitor the platform utilization to identify and solve any digital implementation problem. There is no anticipated harm for trial participation. Participants will receive a 20-euro compensation voucher after accomplishing the program and the two follow-up evaluation protocols.

### Outcome measures

#### Primary outcome measures

##### Children’s dietary intake

Children’s dietary intake is evaluated through an online 24-h food recall developed for this study (*SmartKidsDiet24*). Parents are asked to record all the foods eaten by the child in their presence, foods eaten in meals prepared or offered by parents (e.g., snacks sent to school), or foods that parents are sure that the child ate in the specific days chosen by the app. Foods offered by childcare facilities are not recorded. The app guides parents in registering food and portions for the five main meals (breakfast, morning snack, lunch, afternoon snack, dinner), but it is possible to add an extra meal or snack. Parents also report the time (hh:mm) the food or meal was eaten. The *SmartKidsDiet24* uses an electronic food composition database developed by INSA (National Institute of Health Doutor Ricardo Jorge), compiling several food information sources (i.e., analytical studies, scientific literature, label information, other food composition tables, nutritional composition calculation for specific recipes according to the EuroFIIR method) adapted to the Portuguese diet. To better comply with the intervention aims, the database was updated with sugar-sweetened foods/beverages and other processed foods frequent in Portuguese children’s diet and foods included in vegetarians and vegan diets (e.g., dairy and meat substitutes). The *SmartKidsDiet24* uses the child’s hand as a portion size tool [35] to estimate the food quantities and follows the recommendations for 24-h food recall dietary assessment implementation [36]. Parents are shown which meals have already been registered (e.g., green color instead of gray). When a meal is selected, parents have access to the list of foods previously added to that meal. When adding new foods, parents have two options: search by name (with auto-complete) or by food category. In the end, parents are asked if they want to continue adding others foods to that meal. They are also alerted to remember to add complementary items (e.g., chocolate in the milk). Data regarding the mean number of portions of vegetables, fruit, and sugar-sweetened foods and beverages registered on 3 days will be extracted from the database and analyzed separately as primary outcomes. Time points are as follows: baseline, immediately after the intervention, 3 months after the intervention, 6 months after the intervention.

##### Parental feeding practices

Parental feeding practices are evaluated through the *Food Parenting Practices Questionnaire* [37]. The questionnaire includes three main components: *Promotion of children’s intake self-regulation practices* (e.g., item 9: “If the child says he/she wants to eat more but I think the child had enough, I encourage him/her to stop eating.”), *Food availability and accessibility practices* (e.g., item 23: “I include some form of fruit in most meals*.*”), and *Ineffective control practices* (e.g., item 33: “I withhold sweets/dessert from my child in response to bad behavior.”). Parents answer the 40 items on a 5-point Likert scale (from *Totally false* to *Totally true*). Higher values on each scale indicate more frequent use of each type of practice. In an online study with parents of 2- to 5-year-old Portuguese children, the parental feeding subscales’ internal consistency ranged between 0.65 and 0.89, with an inter-item correlation mean (IICM) between 0.30 and 0.74 [37]. The three scores regarding *promotion of children’s intake self-regulation practices*, *food availability and accessibility practices*, and *ineffective control practices* are studied as primary outcomes. Time points are as follows: baseline, immediately after the intervention, 3 months after the intervention, 6 months after the intervention.

#### Secondary outcome measures

##### Parental perceived barriers related to food and feeding

Parental barriers are evaluated through the *Parental Perception on Children’s Healthy Feeding Barriers Questionnaire* [37], also developed for this study. The instrument includes 30 items organized in five subscales (*Child-related barriers*, *Parent-related barriers: vegetables & fruit*, *Parent-related barriers: added sugars*, *Context-related barriers, Cost-related barriers*). Parents answer on a 5-point Likert scale (from *Totally false* to *Totally true*). Higher values on each subscale indicate that the feeding barrier is more frequently identified. The subscales showed internal consistency scores between 0.66 and 0.95 and IICM between 0.25 and 0.90 [37]. Time points are as follows: baseline, immediately after the intervention, 3 months after the intervention, 6 months after the intervention.

##### Food parenting self-efficacy

To access food parenting self-efficacy, we used the *Parental Self-efficacy for Children’s Healthy Diet Scale* [38, 39]. The questionnaire aims to assess the extent to which parents are sure about their ability to promote the child’s intake of healthy foods and control the child’s intake of unhealthy foods, with four items answered on a 5-point Likert scale (from *No sure* to *Absolutely sure*). Higher values correspond to higher parental self-efficacy. The internal consistency of the instrument is acceptable (*α* = 0.74; IICM = 0.35) and has a good test-retest reliability (rs = 0.78, *p* > 0.01) [38, 39]. Time points are as follows: baseline, immediately after the intervention, 3 months after the intervention, 6 months after the intervention.

##### Parental motivation to change

Parental motivation to promote healthy changes in the child’s diet and parental feeding practices is measured through a set of three items answered in a 10-point numerical scale developed for this study and adapted from Rollnick et al. [40]. Each item evaluates a specific component of parental motivation: (i) the *importance* of participating in a program to help parents promote healthy eating patterns in children, (ii) the *confidence* to perform the proposed tasks and maintain engagement in the program, and (iii) the *readiness* to make changes in the child’s diet and their feeding behaviors. Higher mean scores indicate a higher motivation to change. The instrument showed an acceptable internal consistency (*α* = 0.76) in an intervention study with the ACT program that promoted positive parenting practices of children up to 8 years [41]. Time points are as follows: baseline, immediately after the intervention, 3 months after the intervention, 6 months after the intervention.

#### Other study variables

##### Sociodemographic information

Several parent and child variables are collected in the sociodemographic questionnaire (i.e., parents’ age and sex, level of education, kinship with the child, number of children and adults in the household, country of residency, and if parents receive child benefits). Parents also report specific information about their child: birth date, sex, childcare attendance, current professional support due to weight or eating problems, and the existence of chronic health or food intolerances and allergies. Time point is baseline.

##### Parent’s and child’s weight and height

Both parents’ and children’s weight and height measurements are self-reported. To increase parents’ report accuracy, the app shows specific instructions about assessing weight and height (e.g., in light clothing) correctly. The child’s BMI and percentile are calculated according to the WHO Child Growth Standards (BMI for age and sex). Time points are as follows: baseline, immediately after the intervention, 3 months after the intervention, 6 months after the intervention.

##### Perception of the child’s weight

Parents are asked to rate their child’s current weight subjectively, considering their age and height (underweight, average weight, overweight). Time point is baseline.

##### Concerns about the child’s weight

This dimension is assessed with the *Concern about the Child Weight* subscale of the *Child Feeding Questionnaire* — *Revised* [42] in its Portuguese version [43] and an additional question (i.e., *Considering your child’s height and age, please rate your concern about your child’s current weight*). All items are answered on a 5-point Likert scale (from *No concern* to *Very concerned*). Higher values in the subscale/item correspond to greater concerns about the child’s weight. The internal consistency of the Portuguese version was good (*α* = 0.87) [43]. Time point is baseline.

##### Child’s temperament

The temperament assessment was based on previous studies that identified several temperament clusters in preschool-aged children samples [44–47]. We retained the three temperament styles that seemed more consistent between studies: poor self-regulated or under controlled, inhibited or reactive, and easy or well-adjusted. Parents read three sentences that describe those temperament types and are asked to identify which better describe their child. Time point is baseline.

##### Application usability

To assess usability, we will use the System Usability Scale [48]. It is a commonly used, validated 10-item questionnaire that asks users to rate a system on a 5-point Likert scale from “1 = strongly disagree” to “5 = strongly agree.” Questions focus on the ease of use of the system and the integration of various functions within it. Time point is immediately after the intervention.

### Process evaluation

One of the benefits of a digital approach is the ability to instrument the application with logging capabilities, enabling quantification of usage, usability, and engagement. Our solution will be storing a record of timestamped user interactions, which would ultimately enable us to replay a timeline of actions. We are particularly interested in analyzing the number of accesses to the application and overall time; date and duration spent in each session, resource, and task; quality of the interaction in the interactive activities; the number of clicks and pages visited per session; and the number of notifications and time from notification to engagement.

### Data management

Considering that the RCT design includes repeated measurements, participants’ identification must be recorded to match data across time points. Data regarding the parents’ evaluation protocols, self-monitoring, or interaction with the app are associated with personal accounts. Only information relevant to the study is collected. Only one IT team researcher will access participants’ personal data (collected in the account registration) and unique identification codes. Online informed consent protocol includes a paragraph that explains which data will be recorded and how it will be saved. Parents are asked for permission for the research team to access this information, and to share relevant data with people from the Faculties taking part in the research, where needed. This trial does not involve collecting biological specimens for storage. All data will be stored in a password-protected secure study database. The host of the website will be the Amazon Web Services server. When the data collection has finished, the participant’s identification data will be destroyed, and data related to evaluation protocols and consent forms will be stored for 5 years in the same database.

We do not anticipate any problems that are detrimental to the participant and that require performing interim analyses or the definition of formal stopping rules for the trial. The Project Management Group will meet to review trial conduct once a month; a Data Monitoring Committee was not considered for this trial, as this is a low-risk intervention. The trial will be concluded when the estimated number of participants needed for this trial (*n* = 260) is reached. Any changes to the protocol will be notified by the PI to the sponsor and funder. Any deviations from the protocol will be fully documented using a breach report form. We will also update the protocol in the clinical trial registry.

### Statistical analysis

We will use intention-to-treat principles, with the participants being analyzed in the group in which they were allocated in the randomization process, independently of whether they had completed all measurement time points and/or the intervention. First, an evaluation of missing data mechanisms will be performed to inform which imputation strategy should be applied between multiple imputations (MI) and maximum likelihood estimation (FIML). Assuming the data are missing at random, a sensitivity analysis will be included, considering the whole samples vs. samples with complete data separately.

A descriptive analysis of demographic data and outcome variables at baseline will be performed for parents’ and children’s characterization purposes; the samples in each condition group will also be compared for all dimensions studied. Repeated measures analyses will be used to analyze the differences between condition groups and measurement time points over time regarding primary outcomes, adjusting for potential covariates. To study parents’ dropout predictors, we will first assess the differences between completers, early and late dropouts regarding demographic data, and all variables at baseline. The relationships between parents’ and children’s dimensions with dropout rates will be assessed. We will then conduct a binomial logistic regression to predict the overall dropout. The analysis of individual health trajectories regarding parents’ and children’s variables evolution throughout the intervention will be studied using mixed models. The *p* value will be corrected according to the number of primary outcomes considered in each statistical analysis.

### Data dissemination

The findings will be presented throughout the RCT study at national and international scientific meetings in nutrition, clinical and health psychology, childhood, and parenting, and several articles will be prepared and submitted. At the end of the study, we will disseminate the project results in three ways: (i) a national meeting to present the results from the whole project, including evidence-based guidelines to promote parent’s engagement and decrease parental dropout in health-related interventions; (ii) a workshop of evidence-based methods aimed at changing parents’ feeding practices, to be offered to all professionals from the institutions that collaborated in the recruitment for the project; and (iii) a web page with the project’s main results and guidelines for professionals and researchers. Parents who participated in the program can request a final study report, which will be sent by email.

## Discussion

*SmartFeeding4Kids* was designed to be a flexible, cost-effective, and tailored program for parents who want to improve their feeding practices and develop a healthy diet in their young children. The program gives special attention to promoting feeding practices that encourage children’s eating self-regulation (structure and child’s autonomy promotion practices) as developmentally appropriate and effective alternatives to coercive, restrictive, or permissive feeding practices. This web-based intervention is self-guided and includes self-regulation techniques to help parents become more aware of what feeding practices they are using and how frequently and implement changes according to their goals. The strategies to implement new action plans suggested during the sessions reflect the everyday challenges parents face at mealtimes with young children. The program is supported by well-established and empirically validated theoretical models used in previous parenting and nutritional interventions, and the study design is ruled by the best practices from RCTs for non-pharmacological interventions [18, 49]. Combining these main features is innovative, allowing the study of the intervention’s efficacy on both children’s intake of healthy/unhealthy foods and parental feeding practices and the child’s and parent’s change trajectories on these dimensions across time.

During the program’s development, we considered some issues raised by earlier reviews and interventional studies about similar parenting programs and retained strategies and procedures relevant to those programs’ efficacy. We focused on providing information based on scientific evidence and the most current nutritional recommendations regarding healthy eating and positive parenting practices during the preschool years. The multimedia content was prepared by researchers from different professional domains and designed to be appealing, easily understood by participants with varying literacy levels.

Beyond the traditional BCTs commonly presented in health promotion programs, based on the provision of information (e.g., about the consequences of using specific feeding practices or about the steps to apply a feeding behavior in a particular situation), we invested in a constellation of self-regulation techniques that have already proven to be, individually or together, effective in promoting health behaviors [13, 50]. Techniques like self-monitoring, goal setting, or feedback were repeated in all the sessions to maintain a stable framework for adopting new positive feeding practices and keep parents engaged in their commitments. In some programs, parents not always defined objective, reachable, and realistic goals [28], which might compromise the intervention’s efficacy. We systematically reviewed several parental online interventions to promote children’s healthy eating and positive feeding practices and found that parents rarely received feedback about monitoring their behaviors or information about accomplishing the goal set [8]. In our program, a list of goals based on the parents’ needs identified in the baseline guided parents in choosing the feeding behaviors to improve. The achievement of the parents’ goals is validated at the beginning of the next session, according to the parent’s records and their progress along time.

Guidance about forming new feeding habits was included in the program’s last sessions to reinforce context-dependent repetition and behavior automaticity. Habit formation has been included as a behavior change technique in some parental interventions to promote their children’s healthy dietary patterns and household food availability [51, 52]. However, it is not yet clear how this strategy can promote effective parental feeding practices.

Regarding methodological issues, we defined a high-quality study design with a reduced risk of bias, enhancing the internal validity and the possibility of being replicated and compared [53]. The app was developed to allow strict control of the whole process without researchers’ interference once the program is started, guaranteeing a parent’s full individualized and tailored experience. The study protocol was clearly described regarding the components and contents of the intervention, detailing the parental feeding practices and children’s dietary outcomes targeted by the intervention and providing a categorization of the BCTs used in both arms. The power size and the sample size estimation were based on systematic reviews carried out with similar studies [8, 32, 33]. For this calculation, we adopt a more conservative approach to determine the dropout rate, to accommodate some issues found in online parental interventions and further limitations due to the current coronavirus disease (COVID-19) pandemic context. An extended recruitment plan was developed to tackle those obstacles, and a modest monetary incentive for full participation was added. The delivery of an active control condition allows every parent to access some intervention, reducing participants’ attrition.

Possible limitations of our study protocol include the following: We anticipate most participants to be highly interested and motivated parents, with children with fewer feeding issues, possibly with a higher education level. We also expect most participants will be mothers. Although this is a common issue in parental nutrition interventions, children’s dietary patterns can be influenced by the other caregivers’ practices and the overall home food environment. Only the enrolled participant can complete the tasks and answer the questionnaires and food records. Also, all the measurement outcomes are reported by parents, including the objective measures (i.e., 24-h food recall, children’s and parents’ BMI), and those reports rule the self-regulatory strategies throughout the sessions. The RCT’s intervention arm is quite demanding due to the regular monitoring between sessions and the tasks required during the sessions. This experience can be challenging for some overwhelmed, time-constrained, or less motivated parents and might contribute to a high dropout rate. Also, a lack of direct contact with the research team can decrease parents’ involvement in the program. Although a design with an intervention arm and an active control condition comply with high-quality methodological standards, it also can lead to a less-expressive difference between conditions.

## Trial status

This is the first version of the protocol. The recruitment has started on July 13, 2021, and is expected to be completed on May 2022.

## Supplementary Information

**Additional file 1.** Categorization of the behavior change techniques (BCTTv1 taxonomy, Michie et al., 2013) used in each condition of the RCT.

**Additional file 2. **Outcome measurements and cut-offs points to tailor available goals in the *SmartFeeding4Kids* sessions.

**Additional file 3.** SPIRIT 2013 Checklist.

**Additional file 4.** The TIDieR (Template for Intervention Description and Replication) Checklist.
**Additional file 5. **Roles and responsibilities.

## Data Availability

All data related to study protocol is available in the manuscript or as additional files.
