# Supplementary material for: SmartFeeding4Kids, an online self-guided parenting intervention to promote positive feeding practices and healthy diet in young children: study protocol for a randomized controlled trial
Source: Trials. 2021 Dec 18;22:930. doi: 10.1186/s13063-021-05897-z (PMC8683823; doi:10.1186/s13063-021-05897-z)
Supplement: Supplementary file 1 — Additional file 1. Categorization of the behavior change techniques (BCTTv1 taxonomy, Michie et al., 2013) used in each condition of the RCT. [file 13063_2021_5897_MOESM1_ESM.docx]

Additional file 1. Categorization of the behavior change techniques (BCTTv1 taxonomy, Michie et al., 2013) used in each condition of the RCT.

| **BCT category** | **How is the BCT applied in the intervention program?** | ***SmartFeeding4Kids*** | ***Psychoeducational condition*** |
| --- | --- | --- | --- |
| 1.1. Goal setting (behavior) | A list of theme-related goals is presented to parents at the end of each session based on the child’s food intake and the feeding practices outcomes at baseline. The goals are defined in terms of the parental behavior to be achieved and are tailored, i.e., the goals that appear on the list are only about behaviors that do not meet the guidelines (child’s food intake) or are below the cut-off points defined (parental practices). Parents choose two goals in each session and agree to achieve them during the week. | x |  |
| 1.2. Problem solving | In the quizzes, parents are prompted to analyze the vignettes that describe feeding issues discussed in the session and select the best strategies/practices to overcome barriers. | x |  |
| 1.4. Action planning | Parents are prompted to plan how they will accomplish the goals during the week, based on the information provided during the session. The goals available in the list define a specific context, frequency, and/or intensity for the behavior. | x |  |
| 1.6. Discrepancy between current behavior and goal | At the beginning of the sessions, graphs with information about the targeted behaviors (from baseline to the last record made between sessions) are shown to parents. Based on this data, parents are informed whether their goals were met or not. | x |  |
| 2.2. Feedback on behavior | During the program, parents receive three types of feedback relative to their feeding practices and habits: i) descriptive feedback, that is based on the baseline assessment and is given before a specific behavior is discussed for the first time in the session; ii) evaluative feedback when the performance of a behavior is compared with specific guidelines and cut-off points; and iii) comparative feedback, that shows the evolution on specific behaviors along the weeks since the baseline assessment, to draw attention to differences between the parent’s current behavior and the goals set. | x |  |
| 2.3. Self-monitoring of behavior | Between sessions, parents are asked to evaluate and record how frequent was the use of specific feeding practices (related to the objectives previously defined) during the week. | x |  |
| 2.4. Self-monitoring of outcome(s) of behavior | Between sessions, parents are asked to monitor and record the child’s intake of vegetables, fruit, legumes, and sugar-sweetened foods and beverages during a day of their choice using a 24h food intake instrument. | x |  |
| 2.7. Feedback on outcome of behavior | Parents receive descriptive, evaluative, and comparative feedback about the child’s food intake of vegetables, fruit, legumes, and sugar-sweetened foods and beverages during the program. These outcomes are indicators of the performance of specific feeding behaviors (p.e. “Offer every day to my child three portions of fruit.”) and inform about accomplishing the goals available in session 1. | x |  |
| 3.1. Social support (unspecified) | Parents receive tailored support and encouragement from the app when the goals are not accomplished. | x |  |
| 4.1. Instruction on how to perform a behavior | Parents are advised about using specific positive feeding practices as an alternative for coercive practices and towards the child, parent, and context-related barriers. Positive feeding practices are explained in detail and adjusted to specific situations (*What to do when my child refuses a vegetable? What to do if my child asks for more food?*). | x | x |
| 5.1. Information about health consequences | Parents receive information about the negative health consequences of regularly offering sugar-sweetened foods and beverages to the child (p.e., excessive weight, tooth decay), or the impact of coercive practices on the child’s food preferences and dietary patterns. | x | x |
| 5.3. Information about emotional consequences | Parents receive information about how positive feeding practices can contribute to lesser parental concerns about the child’s diet and more pleasant family mealtimes. | x | x |
| 6.1. Demonstration of the behavior | Examples of verbalizations complement the explanations about how to perform effective feeding practices in specific contexts (p.e., *How do you prefer me to cook the rice? With peas or carrots?*). | x | x |
| 7.1. Prompts/cues | During the week, parents receive notifications to remind the set goals and the main messages of the last session. | x |  |
| 8.1. Behavioral practice | In session 4, parents are asked to identify the obstacles that make it harder for their child to eat healthy and the strategies that can help overcome those barriers. During the formation of new feeding habits (session 5), parents are prompted to adopt specific feeding behaviors according to a plan, to be performed consistently during mealtimes to increase the habit. | x |  |
| 8.2. Behavior substitution | Parents are suggested to replace ineffective feeding practices (p.e., pressure to eat, restriction of unhealthy foods) with effective feeding practices (p.e., food availability and accessibility, modeling). | x | x |
| 8.3. Habit formation | Parents are prompted to adopt new habits in their routine repeatedly as a response to a specific feeding context cue (p.e., *At breakfast, offer healthy alternatives, e.g., natural yogurt, white milk, bread, instead of sugary cereals and other foods*) and receive guidance about how to transform a behavior into a habit. | x |  |
| 10.3. Non-specific reward | Parents receive points and badges when the goals are accomplished and when the progress on the feedback graphs is positive. | x |  |
| 10.4. Social reward | Parents receive a tailored verbal reward when the goals are accomplished. | x |  |
| 10.6. Non-specific incentive | Parents are informed that they will receive points and badges when the goals are accomplished, and the feedback graphics show a positive evolution. | x |  |
| 12.1. Restructuring the physical environment | Parents are advised to control the family’s access to unhealthy foods (e.g., not to buy sugar-sweetened foods or have them at home, instead of hiding or having them closed in a cupboard). | x | x |
| 12.2. Restructuring the social environment | Parents are advised to change the way they involve the children in the food preparation and the choice of foods in the supermarket. | x | x |
| 12.3 Avoidance/reducing exposure to cues for the behavior | Parents are advised to change their usual dairy routines if they involve going through places where there are many “sugary temptations”. | x | x |
| 13.1. Identification of self as a role model | Parents are explained that children tend to imitate their parents’ eating behavior. | x | x |
| 13.2. Framing/reframing | It is suggested that parents adopt a new perspective on parent’s and child’s roles regarding food and eating to promote more effective feeding practices. The golden rule *(parents decide about what, when, and how the child will eat; the child decides whether and how much they will eat)* is introduced in session 2 and repeated throughout the program. | x | x |
| 14.4 Reward approximation | Parents are rewarded with points following any approximation towards the defined goals (observed in the feedback graph). | x |  |

Legend: Target behaviors: Parental feeding practices (encompassing a range of behaviors); Target behaviors outcomes: Child’s dietary intake; Target population: parents
