# Supplementary material for: SmartFeeding4Kids, an online self-guided parenting intervention to promote positive feeding practices and healthy diet in young children: study protocol for a randomized controlled trial
Source: Trials. 2021 Dec 18;22:930. doi: 10.1186/s13063-021-05897-z (PMC8683823; doi:10.1186/s13063-021-05897-z)
Supplement: Supplementary file 2 — Additional file 2. Outcome measurements and cut-offs points to tailor available goals in the SmartFeeding4Kids sessions. [file 13063_2021_5897_MOESM2_ESM.docx]

Additional file 2. Outcome measurements and cut-offs points to tailor available goals in the *SmartFeeding4Kids* sessions.

| **Variable** | **Instruments and subscales** | **Cut-off points** | **List of available goals to be chosen** | **Available at session…** |
| --- | --- | --- | --- | --- |
| Child’s dietary intake reported by parents | *SmartKidsDiet24* | < 3 portions of vegetables a day | Offer my child three portions of vegetables every day | 1 |
|  |  | 3 or more portions of vegetables a day | Offer my child, at least, three different vegetables during the week | 1 |
|  |  | < 2 portions of fruit a day | Offer my child two portions of fruit every day | 1 |
|  |  | 2 or more portions of fruit a day | Offer my child, at least two different fruits during the week | 1 |
|  |  | < 1 portion of legumes a day | Offer your child one portion of legumes every day | 1 |
|  |  | 1 or more portions of legumes a day | Offer my child different legumes during the week | 1 |
|  |  | > 1 portion of sugar-sweetened foods and beverages | Reduce the supply of sugar-sweetened foods and beverages during the week | 1 |
| Parental feeding practices | Pressure to Eat | > 2,5 (maximum mean score: 5) | Serve small amounts of food, and expect the child to ask for more if he/she has an appetite.  Allow the child to stop eating if he/she says that is he/she full, that he/she is not hungry, or that he/she does not like that food. | 2 |
|  | Food as a Reward | > 2,5 (maximum mean score: 5) | Offer other rewards (that are not food) when I want to reward the child (read a story, play a game). | 2 |
|  | Giving Healthy Food Options | < 3,5 (maximum mean score: 5) | Involve the child in choosing healthy foods for meals.  Let the child choose what he/she prefers to eat, among healthy options available. |  |
|  | Food Intake Self-Regulation Teaching | < 3,5 (maximum mean score: 5) | Explain to the child why healthy foods are important and are good (e.g., The apple makes your heart stronger! The carrot helps you see better at night, like owls!) | 2 |
|  | Modeling to promote vegetable intake | < 3,5 (maximum mean score: 5) | Be a good role model for the child, eat healthy foods at meals, and show that you like to eat them.  Use dolls and characters that the child likes to show they also eat and like healthy foods. | 2 |
|  | Exposure to Vegetables & Fruits | < 3,5 (maximum mean score: 5) | Have ready-made or easy-to-prepare healthy foods available and accessible.  Encourage the child to know and taste new foods without putting pressure on him/her to eat. | 2 |
|  | Emotional Feeding | > 2,5 (maximum mean score: 5) | When the child is angry, sad, or bothered, talking to the child comfort or distract them without resorting to food. | 3 |
|  | Food Restriction | > 2,5 (maximum mean score: 5) | Establish limits on the intake of unhealthy foods.  Allow the child to eat unhealthy food only on special occasions. | 3 |
|  | Limitation of unhealthy foods availability | < 3,5 (maximum mean score: 5) | Establish clear rules regarding the purchase of unhealthy food or the unhealthy food I bring home.  When going out with the child, bring healthy drinks and/or food in case he/she is hungry. | 3 |
|  | Food Intake Self-Regulation Teaching | < 3,5 (maximum mean score: 5) | Explain to the child what can happen if they eat an excessive amount of food (e.g., *I realize that you like this food a lot. But if you eat more now, you may run out of energy to play*.)  Explain to the child what can happen if they eat many unhealthy foods (e.g., I realize that you like this chocolate a lot, but if you eat more now, *you can be sick*.) | 3 |
|  | Food Intake Self-Regulation Prompting | < 3,5 (maximum mean score: 5) | Ask the child to identify hunger and satiety cues when he/she asks for food between meals (e.g., *How do you know that your tummy is still hungry? What do you feel?*)  Help the child decide whether he needs to eat more or not, based on his/her hunger and satiety cues (e.g., *Let's hear what your tummy tells you. Do you think it is satisfied or asking for food?*) | 3 |
| Feeding habits | Self-Report Habit Index (SRHI): Self-Report Behavioral Automaticity Index | < 5 (maximum mean score: 7 ) | At lunch and dinner, offer a soup of vegetables before the main plate.  At lunch and dinner, offer vegetables on the plate  At lunch and dinner, offer legumes on the plate  At lunch and dinner, offer fruit  At lunch and dinner, offer water  For breakfast, offer fruit  For breakfast, offer healthy alternatives to sweet cereals and other foods with added sugar (bread, plain milk, plain yogurt)  Offer fruit as a snack  Offer healthy alternatives to cakes, juices, and other foods with added sugar (bread, plain milk, plain yogurts, healthy snacks) as a snack  When the child is thirsty, or between meals, offer water | 5 |
